# Gluten and FODMAPs Relationship with Mental Disorders: Systematic Review

**DOI:** 10.3390/nu13061894

**Published:** 2021-05-31

**Authors:** Egoitz Aranburu, Silvia Matias, Edurne Simón, Idoia Larretxi, Olaia Martínez, María Ángeles Bustamante, María del Pilar Fernández-Gil, Jonatan Miranda

**Affiliations:** 1 Gluten Analysis Laboratory of the University of the Basque Country, Department of Nutrition and Food Science, University of the Basque Country, 01006 Vitoria, Spain; earamburu011@ikasle.ehu.eus (E.A.); smatias002@ikasle.ehu.eus (S.M.); edurne.simon@ehu.es (E.S.); olaia.martinez@ehu.eus (O.M.); marian.bustamante@ehu.eus (M.Á.B.); mariadelpilar.fernandez@ehu.eus (M.d.P.F.-G.); jonatan.miranda@ehu.eus (J.M.); 2 GLUTEN3S Research Group, Department of Nutrition and Food Science, University of the Basque Country, 01006 Vitoria, Spain; 3 Bioaraba, Nutrición y Seguridad Alimentaria, 01006 Vitoria, Spain; 4 Centro Integral de Atención a Mayores San Prudencio, 01006 Vitoria-Gasteiz, Spain

**Keywords:** gluten-free diet, low FODMAP diet, clinical trial, randomized controlled trial, depression, anxiety, cognition, Alzheimer’s disease, schizophrenia, autism spectrum

## Abstract

Nowadays, gluten and FODMAP food components (fermentable oligosaccharides, disaccharides, monosaccharides and polyols) are increasingly studied due to their possible relation with extraintestinal-associated conditions. In recent years, gluten-free diets (GFD) and low-FODMAP diets (LFD) are becoming more popular not only in order to avoid the food components that cause intolerances or allergies in some people, but also due to the direct influence of marketing movements or diet trends on feeding habits. Likewise, neurological and psychiatric diseases are currently of increasing importance in developed countries. For this reason, a bibliographic systematic review has been carried out to analyse whether there is a pathophysiological relationship between the dietary intake of gluten or FODMAPs with mental disorders. This review collects 13 clinical and randomized controlled trials, based on the PRISMA statement, which have been published in the last ten years. Based on these results, limiting or ruling out gluten or FODMAPs in the diet might be beneficial for symptoms such as depression, anxiety (7 out of 7 articles found any positive effect), or cognition deficiency (improvements in several cognition test measurements in one trial), and to a lesser extent for schizophrenia and the autism spectrum. Nevertheless, further studies are needed to obtain completely reliable conclusions.

## 1. Introduction

Gluten is the storage protein of wheat and is made up of a complex mixture of proteins, mainly gliadin and glutenin. Other cereal storage proteins such as rye, barley or oats are also referred to as gluten. Gluten-containing cereals are staple foods, but due to its ability to act as a binder and extender, gluten is also added to processed food to improve its texture and moisture characteristics [1]. One of the components of gluten is gliadin, which contains a series of peptide sequences with a high resistance to proteolytic digestion in the gastrointestinal tract. This quality comes from the large amount of proline and glutamine amino acids contained in gliadins, which are only slightly hydrolyzed by proteases [1,2]. Gluten is one of the most common factors in inducing gastrointestinal symptoms, as is historically and consistently demonstrated [3]. It is also responsible for the immunogenic response in celiac disease (CD).

This illness causes mucosal inflammation, atrophy of the intestinal villi, increased intestinal permeability, eventual nutrient malabsorption and subsequent nutritional deficiency. The only treatment for CD is to follow a strict lifelong gluten-free diet (GFD) [4]. This food restriction is also applied to treat other disorders than CD, in which gluten proteins can cause symptoms in patients, such as non-celiac gluten sensitivity (NCGS). Due to variable symptoms, both gastrointestinal and non-intestinal, NCGS involves a heterogeneous group of patients [5].

Nowadays, a considerable amount of not-celiac people avoid gluten in their diet based on the widespread belief that a GFD brings health benefits. This GFD trend, as well as food industry influence, leads many people to consume gluten-free products unnecessarily [6]. Other food components can be limited or avoided in CD or NCGS treatments, such as fermentable oligosaccharides, disaccharides, monosaccharides and polyols, known as FODMAPs [7]. These include fructose in excess of glucose, lactose, fructans and galactooligosaccharides (GOS), sorbitol, mannitol, xylitol and maltitol. These compounds share some functional properties such as poor absorption in the small intestine, osmotic activity and fast fermentation by bacteria [8,9,10,11]. Focusing on NGCS, both wheat-, gluten- and FODMAP-containing foods may play an important role in the immune response [12,13]. Therefore, some authors proposed the term non-celiac wheat sensitivity [14].

As these compounds have been defined recently, less information on FODMAP-containing foods is available compared with gluten. High FODMAP-containing foods can be found in staple foods of the western diet, such as fruits and vegetables, milk and dairy products, legumes and cereals. They are also found in wheat and rye [15], so the consumption of these foodstuffs or their derivatives entails both gluten and FODMAPs combined intake. FODMAPs can induce abdominal bloating, pain, vomiting and alteration of stool frequency, especially in those suffering from irritable bowel syndrome (IBS) [16,17]. Apart from gastrointestinal symptoms, other extra intestinal clinical manifestations have been attributed to the FODMAP and gluten combination. In this regard, dermatological disorders, such as dermatitis, rashes and eczema diseases have been detected in both CD and NCGS [18,19]. In addition, CD has been associated with other autoimmune diseases, such as type 2 diabetes, autoimmune thyroiditis and dermatitis herpetiformis [20], and similar symptomatology could be ascribed to NCGS. Furthermore, rheumatological disorders and autoimmune disorders have been associated with wheat consumption, although evidence is lacking [21].

Several studies have explored the relationship between gluten intake and neurological and psychiatric alterations in symptoms such as ataxia, peripheral neuropathy, schizophrenia, autism, depression, anxiety and hallucinations [22]. Ataxia, neuropathy and encephalopathy are the most frequent [23]. Among mental diseases, it is hypothesised that NCGS may be associated with anxiety and depression [24], as well as with schizophrenia and bipolar disorder [25]. The above-mentioned relationship between wheat and autism is under revision [26].

The connection between gluten or FODMAPs and the central nervous system (CNS) may occur through mechanisms such as intestinal permeability and intestinal dysbiosis. In addition, the blood-brain barrier (BBB) could be adversely affected [27,28]. Indeed, in macaques induced with CD, a decrease between the tight junction of enterocytes and altered expression of their modulating proteins was observed. Moreover, microbiota imbalance may play an important role in the pathogenesis of CD, which would justify the aforementioned dysbiosis [28]. This may facilitate the passage of microorganisms and derivatives into systemic circulation. Long-term translocation may have an effect on hepatic clearance, increasing the potential concentration of lipopolysaccharides. These molecules can cross the BBB and activate microglia cells, leading to neuroinflammation and neuronal damage [29]. In addition, metabolites from the microbiota in dysbiosis may contribute to post-translational modification of proteins such as transglutaminase (TGM), which are important in CD, regulating the functioning of the gut-brain axis [30].

Other factors such as extracellular structures (ES) can affect the CNS, since these structures can regulate the cell biochemical environments and physiology of the organism. One of the archetypes of ES, the membrane-encased extracellular vesicles (EVs) [31], can damage the BBB. Although the mechanism is unknown, EVs contribute to metal transport protein release, restructuring of the outer cellular matrix, epithelial barrier regulation and immune cell sequestration [32]. Indeed, patients with IBS have shown increased levels of EVs [33], which are potentially related with CD [34].

The present systematic review assesses the relationship between gluten and FODMAP intake and some mental symptoms and illnesses that affect the CNS. Bearing this purpose in mind, this work has evaluated the potential benefits of controlled dietary intervention of gluten and FODMAPs on mental disorders, depression, anxiety, schizophrenia and autism spectrum (AS), as well as neurological Alzheimer’s disease (AD). The evidence obtained would sustain the utility of GFD or low FODMAP diets (LFD) to prevent, treat or help in these mental disorders.

## 2. Materials and Methods

PubMed database was used for systematic review of the topic following preferred reporting items for systematic reviews and meta-analysis (PRISMA) guidelines [35]. The terms “gluten diet” [all fields] or “FODMAP diet” [all fields] and “depression” [all fields] or “anxiety” [all fields] or “schizophrenia” [all fields]; “Alzheimer” [all fields] or “autism” [all fields] were included as keywords in the search. The last search was conducted on 30 March 2021.

Results were limited to the last ten years of English published clinical trials or randomized controlled trials, including dietary intervention exclusively related to gluten and FODMAP in human populations. Reviews, editorials, conference papers or abstracts were excluded. Due to the lack of clinical trials related to AD, the search term was widened to “cognition” as the keyword. The reference lists of included articles were screened manually for additional studies by three independent authors (E.A., J.M. and E.S). Disagreements were solved by consensus.

On 20 April 2021, a specific search of ongoing clinical trials was performed according to the previously reported keywords in the clinical trial register (clinicaltrial.gov).

Preclinical studies based on the Mohan et al. review [31] and other individual preclinical studies were used to set the potential mechanisms that link FODMAP and gluten diet with the analysed metal disorders. In the particular case of FODMAP (lactose, fructose, stachyose, raffinose, fructans, sorbitol, or mannitol, the most important FODMAPs according to cut off values), an independent search was performed to establish any relationship with the assessed metal disorders.

## 3. Results

### 3.1. Identification and Selection of the Included Articles

**Figure 1:**
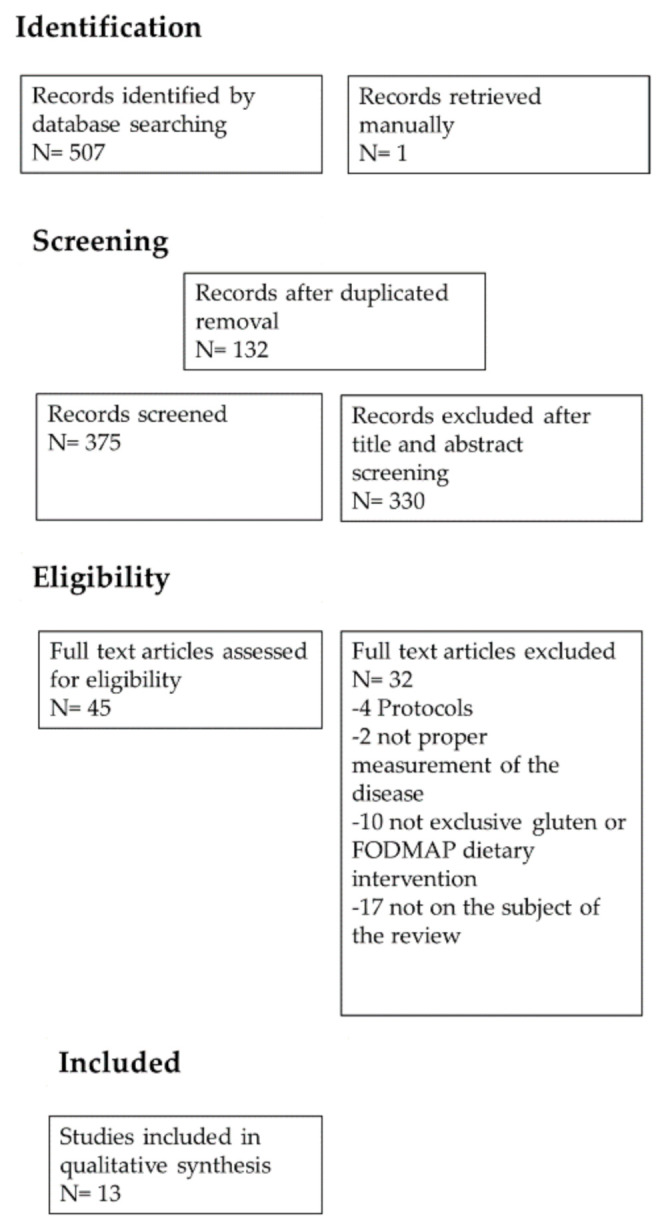
PRISMA flow chart: summary of evidence search and selection.

A summary of the study selection process is shown in Figure 1. A total of 551 articles were identified through PubMed since 2011. After removing duplicates, 375 articles were examined. In a following step, 328 articles were excluded because the abstract or title did not match with the limitation explained in the material and methods section. Of the 47 full text articles assessed for eligibility, 33 were discarded due to the following different motives: being protocols, not being exclusive gluten or FODMAP dietary interventions (increasing or reducing the amount of these dietary compounds), not being the subject of the review (involving metal illness) or lack of quantitative measurements (Figure 1).

### 3.2. Characteristics of Included Studies

The 13 studies included in the current review (Table 1 and Table 2) comprised a total number of 526 patients. Of all the patients, 229 were suffering from IBS, 111 from CD or NCGS, 75 from fibromyalgia, 16 from schizophrenia and 175 from AS. The vast majority of the trials were conducted with adults; only those that were focused on the AS were carried out with children. In the case of adult studies, the age range was from 18 to 75 years, although age range was not described in two trials, where authors only provided a mean age [36,37].

Children from 3 to 6 years old took part in two of the AS studies; in the other one, ages ranged from 6 to 17 years old. Of the 13 selected studies, the female proportion was higher than the male in 7. By contrast, there were more males in 4. In one article, the female to male ratio was not declared. With regard to geographic distribution, 5 studies were conducted in Europe (Poland, Spain, Finland, Italy and United Kingdom), 1 in Asia, 4 in The United States of America and 3 in Australia (Table 1 and Table 2).

Of these 13 studies, 2 were randomized double-blind crossover designs, 3 were single-blind, 4 were not-blinded and 1 was longitudinal in design. Most of the studies evaluated the effect of a GFD, whereas only 3 studies involved the LFD.

In the case of depression and anxiety, the tests most used were: Spielberg State Trait Personality Inventory (STPI), Hospital Anxiety and Depression Scale (HADS) and State (information involving current emotional state) and Trait (information involving emotional disposition) Anxiety Inventory (STAI). In the case of schizophrenia, the Brief Psychiatric Rating Scale (BPRS), Scale for the Assessment of Negative Symptoms (SANS) and other tests where used. Finally, in the case of the AS, Social Communication Questionnaire (SCQ) and Autism Spectrum Rating Scale (ASRS) were used, among other tests. All scales, indices and tests are summarized in Table 1 and Table 2. One of the tests used for anxiety was not validated [37]. A comparison among intervention and control groups was performed in most of the studies, whereas only 3 studies compared pre- and post-intervention variables. Moreover, 8 studies combined both comparisons.

## 4. Discussion

Articles that link gluten- and FODMAP-containing foods with each mental symptom or illness have been analysed and classified individually according to their pathology, in the following order: depression, anxiety, AD, schizophrenia, and AS. All of the articles collected reported human clinical trials (in order to perform a representative analysis). Some characteristics of the studies were taken into account to assess their quality and facilitate comparisons between them (number of participants, age, sex and duration of the intervention).

### 4.1. Depression and Anxiety

According to the fifth edition of the Diagnostic and Statistical Manual of Mental Illness, major or unipolar depression is characterised by one or more major depressive episodes [48]. A major depressive episode consists of a lack of interest or pleasure in all activities for at least two weeks. However, four other symptoms must also be present: changes in appetite, weight, sleep and psychomotor activity; reduced energy; tendency to feel useless or guilty; difficulty in thinking, concentrating or making decisions; repetitive thoughts of death or suicidal ideation. World Health Organization data confirmed that depression is a common mental disorder, with a global prevalence of 4.4%. Furthermore, it must be taken into account that this illness provokes close to 800,000 suicide deaths per year [49].

Anxiety disorder is the term for various forms of a type of mental illness characterised by anxiety, fear and associated behavioural changes. While anxiety is the response to prevent a future threat, fear involves an emotional response to an immediate threat. These responses can produce physical symptoms such as tachycardia and tremors. There is a wide range of anxiety disorders which includes general or social impairments, panic or phobias [50]. The estimated total number of people living with anxiety disorders in the world is 264 million, which means a global proportion of 3.6% [49].

#### 4.1.1. Gluten and FODMAP Intervention Trials in Depression and Anxiety

Seven articles about depression and/or anxiety have been analysed in this work. Trials involving gluten and FODMAPs dietary intervention are cited below.

The first trial assessed was conducted with twenty-two patients, including five males, who suffered from IBS but did not have CD [24]. Patients aged between 24 and 62 years underwent a randomized double-blind crossover trial to determine whether gluten can affect depression. The experimental design included three different diets: I—gluten diet (16 g/day), II—whey protein diet (rich in protein which was considered equivalent to gluten protein) and III—placebo diet. Each participant followed one of these diets for three days and then underwent an up to fourteen-day washout period before moving on to the next diet. Depression was measured using the STPI test (in addition to depression, STPI also assesses anxiety). Participants following the gluten-containing diet showed significantly higher scores for depression than those with the placebo-diet. Based on the data, the mean value of depression in the gluten-diet group was 21.45, compared with 19.20 and 19.75 for the placebo-diet and whey-diet groups, respectively. Seventy percent of the participants showed higher depression scores with gluten; however, no significant differences were found when comparing the gluten-diet group with the whey–diet group. Basic studies in rats and humans reported that the type of protein intake influences levels of the essential amino acid tryptophan, a precursor of serotonin, and thus can modulate the symptoms of depression [51,52]. Apart from serotonin neurons, other kinds of neurons, such as catecholamines, are also involved in mood symptoms [53]. Following this research line, rats fed a single meal of seventeen percent gluten or lactalbumin (present in whey) proteins showed different patterns of response on tryptophan serum concentrations and regional brain serotonin synthesis rates [54]. A lactalbumin-based diet elevated serotonin serum concentration as well as hypothalamic and hippocampal serotonin synthesis, while a gluten-based diet decreased serotonin serum concentration without effect on regional brain serotonin synthesis.

By contrast, the same research pointed out that none of the protein-based diets were able to change the catecholamine synthesis rate. Taken together, it may be thought that due to the poor intake or to the short length of intervention in the twenty-two patient clinical study, gluten promoted a partial worsening in depression symptoms by altering the tryptophan-serotonin pathway. Along the same lines, Di Sabatino et al. [37] enrolled a group of sixty-one suspected NCGS patients (eight of them were men). They had two groups follow different diets: I-gluten containing diet (4375 g/day) and II-placebo diet for a week. After a 1-week gluten-free diet, participants crossed over from one diet to the other. A non-validated rating scale depression questionnaire and STAI test was carried out before and after the intervention. Contrasting results after the intervention with baseline, depression score increased in both groups. Nevertheless, when comparing them, results showed that the gluten-containing diet group presented worse depression symptoms than the placebo group that did not present any.

Aziz et al. [36] studied the effects of a GFD on patients with IBS and diarrhoea. Psychological changes were measured by the HADS. This scale is a highly reliable questionnaire which is divided into fourteen sections; seven of them are specific for the assessment of depression and seven more for anxiety (lower scores indicate a lesser severity of the illness). After following a strict GFD for six weeks, HADS score decreased in a group of forty-one participants. Additionally, the authors described that GFD effect remained after eighteen months following this diet.

The effect of GFD intervention was also evaluated in patients with fibromyalgia [40]. Specifically, in a trial with seventy-five participants, patients were separated into two groups to follow a GFD or a hypocaloric diet for six months. Beck Depression Inventory-II (BDI-II) and STAI tests were used as assessment tools before and after the intervention. Results showed that people on a GFD scored lower points on BDI-II after the intervention. However, no significant differences were shown in the STAI test results. Comparing both groups, no differences were found.

Anxiety was also addressed in another trial with forty patients (twenty-six men) who were asymptomatic and seropositive for CD antibodies. Its design was single-blinded and randomised, and patients were aged between 21 and 74 years [38]. They were divided into two groups following specific diets for one year: I—GFD and II—normal amount of gluten in the diet. After this period, the design was cross-checked. Reduced anxiety symptomatology was observed in the GFD group (*p* = 0.025), taking into account the Psychological General Well-Being (PGWB) score, which is a 22-item quality of life questionnaire for the assessment of psychological well-being, expressed as a summary score [38,55]. As authors exemplified with the results of two patients following each diet, total PGWB score improved only after GFD intervention (diminished in the case of normal diet score).

In general, the studies involving gluten with mood changes can be divided into two large groups: short-term and medium- to long-term studies. There are two short interventions (3 and 7 days) gathered in this review: one with a high dose of gluten (16 g/day) [24] and the other one with a low dose of gluten (4375 g/day, an amount equivalent to two slices of bread) [37]. In both cases, the interventions present similarities that are worth commenting on. Both are trials with crossover interventions and short wash-out periods not exceeding 14 days, which may be a limitation. These studies were carried out in non-celiac adult patients with intestinal problems (IBS or suspected gluten sensitivity). Findings from short-term gluten interventions suggest that this protein worsens depression symptoms in patients. The clear observation of an effect can serve partially to confirm that the sample size greater than or equal to 22 per studied group was sufficient.

Regarding medium or long interventions, the first relevant aspect is that instead of gluten exposure, the validity of a GFD was always tested. The smallest group sample size used was 20 people [36,40], similar to that used for short-term interventions. Although in this case trials were also carried out with adult patients with mean ages of 40, 52 and 42 years, and suffering from IBS [36], celiac disease [38] or fibromyalgia and a degree of gluten sensitivity [40]. Therefore, although it is broadly demonstrated that GFD has a common characteristic of improving depression and/or anxiety, it is convenient to limit the results to the certain type of patient. Thus, by giving continuity to the results with short-term interventions in IBS patients [24], the follow-up of a GFD for 6 weeks or 18 months reduces anxiety and depression values measured by HADS [36]. It is remarkable that this study did not have a control group following a regular diet and, consequently, the obtained outcomes are gingerly considered. In the case of the trial with celiac patients, it did include a group with this type of diet [38]. After 12 months of intervention, improvements in anxiety were observed, but not in the case of depression.

Finally, the study by Slim et al. [40] was carried out with patients with fibromyalgia who also confirmed a minimum of 5 out of 14 symptoms of gluten sensitivity. At the end of 6 months, the GFD group showed an improvement in depression symptoms (BDI-II test), but not in anxiety (STAI). These interesting results are in contrast with findings in celiac patients. Nevertheless, it is highlighted that the fibromyalgia patients allocated in the GFD group did not show significant differences in anxiety or depression compared with the group that followed a hypocaloric diet. This effect may be due to the fact that following both diets (GFD and hypocaloric) entailed a similar reduction in gluten sensitivity symptoms (least squares mean −2.44 vs. −2.13) [40]. This could suggest that, beside the mechanism of action detailed in the following section, the improvement of gastrointestinal symptoms in those patients promotes an increase in mood.

As for FODMAP-containing diets, Peters et al. conducted a trial in seventy-four adult patients (14 of them were men) suffering from IBS [39]. They were divided into three groups: I—who received hypnotherapy (a hypnosis-based healing method used for the treatment of certain diseases), II—who adopted a LFD diet and III—who received a combination of both. Participants in group II were asked to limit foods with high and moderate amounts of all types of FODMAPs and to consume only low or non-FODMAP foods, after being given information about them. Psychological changes were measured at six weeks and six months using the STPI and HADS questionnaires. The contrast between final results with those at the beginning of the trial showed an improvement of the symptoms in the three groups, with no significant differences between them. Regarding the STPI test, there was a significant improvement in the frequency of depressive events in patients within the hypnotherapy group. The same results were observed by the HADS questionnaire, noting that the hypnotherapy group improved these parameters even more than other groups. In fact, the hypnotherapy group continued showing beneficial results from the 6th month onwards, while it kept slowing down in the other groups. In short, the improvement observed when comparing with initial data led the authors to propose that the LFD had beneficial effects.

In another study with ninety-two adults (between 19 and 75 years old; sixty-five women) suffering from IBS and diarrhoea [41], participants were randomly assigned to groups for four weeks: LFD or healthy diet (as recommended by the National Institute for Health and Care Excellence of England). Psychological changes were measured by HADS. Anxiety scores decreased in the LFD group compared with the healthy diet group, indicating a significantly greater enhancement for the first group. In the case of depression, despite scores improving in both groups compared with baseline, this only reached significance in the low-FODMAP group. In addition, anxiety was also assessed. Data from patients on a LFD showed a reduced anxiety score (from 9.13 to 7.73), an effect not found in the modified diet group (from 9.31 to 9.54). Signs of improved anxiety may also include enhanced quality of life, psychosocial characteristics, work productivity and sleep quality, as shown by the results of participants in the LFD group [41].

Even though there were only two studies that related FODMAPs to depression and/or anxiety, both confirmed that interventions with a short duration LFD (4 and 6 weeks) diminished symptoms of depression and/or anxiety, measured with HADS in patients with IBS. In both cases, the methodology used was adequate, with a correct number of patients in the treated group (24 and 50 people), and a close age (mean age 34 years and 42 years). However, two important facts should not be taken lightly. First, in the study by Peters et al. [39] the LFD did not produce changes in anxiety or depression according to the STPI scale, but it did using HADS. This fact could give rise to doubts as to the selection of the method or scale used for its assessment in clinical practice, when a LFD follow-up is recommended. Second, in the clinical trial carried out by Eswaran et al. [41], when a LFD intervention was compared with a healthy diet, the magnitude of the improvement was significantly greater for anxiety, while this outcome was not reached for depression (95% CI, −0.70 to −1.30). This isolated result could be considered circumstantial, but in the study by Peters et al. [39] the lack of effect was also confirmed when the intervention in IBS patients was prolonged for 6 months, based on the HADS. Therefore, it could be concluded that following a LFD is more effective against anxiety than depression.

In conclusion, results obtained in these studies showed positive inferences limited to patient type and length of intervention. From the seven trials analysed, the avoidance of gluten and limitation of FODMAP consumption amended, at least partially, depression/anxiety symptomatology in all of them. Furthermore, according to the clinical trial register, there are four more ongoing trials with gluten intervention (NCT04219813, NCT03027492, NCT04274374 and NCT03664531) and three with FODMAP intervention (NCT04364750, NCT03664531 and NCT04830410), with the aim to evaluate anxiety or depression.

#### 4.1.2. Potential Mechanism of Action in Depression and Anxiety

The aetiology of depression is unclear, but the mechanisms of action of antidepressants currently used in therapy are strongly related to the monoaminergic theory. The deficiency of certain monoaminergic neurotransmitters (serotonin, noradrenaline and dopamine) could be responsible for depressive features [56], and the effectiveness of antidepressants is based on increased neurotransmitter levels. Furthermore, it has been evidenced that major depression is accompanied by an activation of the inflammatory response [57]. According to this pathway, the use of antidepressants such as fluoxetine may normalize pro-inflammatory cytokines levels like IL-6 [58]. Additionally, major depression is accompanied by autoimmune responses which could be explained by an increased gut permeability [57].

Different genes and/or receptors have been postulated as biomarkers, considering the role they play in the course of the depressive symptoms. One of them might be transglutaminase 2 (TGM2). This group of enzymes, not only catalyses the reaction that forms the three-dimensional network produced by protein aggregation (gluten among others), but also serotonin transamidation reactions. Consequently, increased TGM2 (and, thus transamidation as well) decreases the availability of serotonin [59]. Pattern recognition receptors, including NOD2, are a family of receptors that trigger the immune response in the case of tissue damage or microbial infection. Expressed in the gut and brain, NOD2 ensures the proper functioning of the gastrointestinal system by regulating serotonin neurotransmission [60]. The P3 Forkhead box (FOXP3) biomarker plays an important role in the development and function of regulatory T cells, which contribute to the elimination of inappropriate immune responses [61]. Some preliminary proposals suggest that regulatory T cells may contribute to immune imbalance due to their interaction with the serotonergic system [62]. In connection with hippocampus, notch receptor 1 (NOTCH1) and peroxisome proliferator-activated receptor (PPARγ) genes seem to be involved in neurogenesis processes. Basic experimentation with animals confirmed the involvement of these genes with symptoms of depression and anxiety [63,64].

It has been observed that gluten-producing symptoms in CD can cause changes in the levels of the above-mentioned biomarkers, raising the suspicion that gluten is involved in the pathophysiology of depression. Thus, in a trial using duodenal biopsy samples from celiac patients with severe histopathological lesions, NOD2 and FOXP3 levels were altered [65,66]. In paediatric CD, NOTCH1 receptor was found to be reduced and TGM2 levels were also altered [67,68]. In addition, PPARγ expression was reduced in celiac patients, as previously described in the duodenal epithelium of celiac macaques [69,70].

Moreover, it is undeniable that the relief of gastrointestinal symptoms after following a GFD can improve the mood of patients. Therefore, in addition to the aforementioned mechanism of action, this helps to explain the reported reduction in symptoms of depression and anxiety with the GFD. Accordingly, each analysed trial identified a certain type of improvement (bloating, pain, wind, stool consistency or nausea among others) in gastrointestinal symptoms. For instance, after measuring the gastrointestinal symptoms in individuals at risk for CD, Kurppa et al. [38] reported a significant reduction in Gastrointestinal Symptoms Rating Scale scores (difference in mean change −0.4) after one year in a GFD as well as an enhancement in an anxiety scale (difference in mean change 0.6).

With regard to FODMAPs, the mechanisms involved with depression remain unclear. It is generally accepted that carbohydrate intakes and their intestinal absorption facilitate tryptophan synthesis in the brain through insulin activity [71]. Nevertheless, it seems that fructose malabsorption is associated with decreased plasma L-tryptophan, a precursor of serotonin [72]. It has been proposed that availability of amino acids related to depression symptoms, such as tryptophan, could be boosted after a decrease in the fructose dose [73].

Contradictory results were obtained when trying to extrapolate this effect to other FODMAPs. On one hand, Varea et al. proposed that both lactose and fructose were able to interact with tryptophan, resulting in non-absorbable gastrointestinal complexes [74]. On the other hand, a recent study concluded that fructose malabsorption was significantly associated with depression, whereas no relationship was found for lactose malabsorption [75].

The association between the CNS and intestinal barrier dysfunction has been extensively reviewed. The release of pro-inflammatory molecules by intestinal immune cells acting as neuroactive mediators has been proposed as one of the action mechanisms in stress and depressive episodes [76]. In this line, experiments carried out with rats confirmed that a high intake of FODMAPs causes a polysaccharide-mediated inflammation that increases the expression of pro-inflammatory molecules (IL-1β, IL-6, IL-17, TNF-α, and IFN-γ) [77].

In anxiety disorders, the neurotransmitter gamma aminobutyric acid (GABA), (the main restrictor of the activity of the CNS), is known to influence mental symptomatology. Indeed, a large number of anxiolytics are effective in modulating GABA receptors [78]. The most important parts of the brain involved in anxiety are the amygdala and the hippocampus. The amygdala is responsible for physiological responses to an external factor, but it needs the hippocampus because it controls study and memory and links familiar stimuli to fear. Regarding its implication with the hippocampus, NOD2, like NOTCH1, may be related to anxiety, as in the case of depression. Indeed, in NOD2 knock-out mice, anxiety behaviours were observed with changes in serotonergic release leading to hyperactivation of the hypothalamic-pituitary-adrenal axis [60].

With reference to FODMAPs, it is worth mentioning a recent review which focused on the role of the gut microbiota in dietary interventions for depression and anxiety [79]. Its authors postulated that due to the similarities between anxiety/depression and IBS illness’ mechanism of action, LFD could lead to the reversal of dysbiosis related to altered mood. However, they also had concerns about possible negative effects of this diet on microbiota: reduction of total bacterial abundance, potential reduction of beneficial short-chain fatty acids (SCFA), decrease in Bifidobacteria and other beneficial bacteria.

Interestingly, it has also been documented that fructans can affect anxiety and tension [80]. In a study in which an infusion of glucose or fructans was carried out in the stomach, it was observed that between healthy patients and those with IBS, there were differences in psychological parameters. Thus, results related to anxiety showed a slower decrease with fructan perfusion than with glucose (*p* = 0.011). It was also observed that in patients with IBS, the tension level dropped less following fructans compared with glucose (*p* = 0.027). 

It was previously reported that GOS and fructooligosaccharides (FOS) have an effect on anxiety (but not on depression) compared with a placebo [81,82]. The mechanism of action proposed for these FODMAPs was a change in gut microbiota; however, other studies have postulated that a longer time is needed for an improvement in depression than in anxiety, which takes place with medication [83]. The two LFD-trials included in the current review also described similar results, showing that FODMAP influences anxiety more than it does depression [39,41]. Nevertheless, in the case of total FODMAPs, time-length cannot be considered the sole limiting variable, since anxiety and depression scales in HADS survey were modified with four and six weeks of LFD intervention, while anxiety was modified with six months of intervention. By contrast, the test chosen to assess both mood symptoms might be a key aspect; no changes in anxiety and depression for STPI state or trait indices were observed with LFD [39].

### 4.2. Alzheimer’s Disease

Dementia is a term used to describe a clinical syndrome of progressive cognitive decline that interferes with the ability to function independently. According to the cause of the dementia, four subtypes are currently defined. AD is the most common form of progressive dementia. AD is a neurodegenerative disorder characterised by intracellular neurofibrillary tangles and extracellular β-amyloid plaques. The common signs are personality changes, impaired movement, communication difficulties, memory loss, mood changes and attention and orientation problems [84].

Lebwohl et al. carried out a large cohort study to evaluate the risk of dementia in patients with histologically diagnosed CD [85]. Although CD patients are not at increased risk for dementia overall, the authors found an increased risk of vascular dementia among this collective [85]. Even if there are many basic studies that, in theory, associate gluten or CD and AD, information linking them derived from applied research is scarce. This explains the lack of clinical or randomized controlled trials included in the present review. Considering that cognitive impairment is closely related to AD, the only trial including gluten or FODMAP intervention and cognition assessment is described. Specifically, the trial reported data about a milder form of cognitive weakening, namely “brain fog”. This term includes transient cognitive impairments in memory, attention, executive function, and the speed of cognitive processing as defined by others [86].

#### 4.2.1. Gluten and FODMAP Intervention Trials in Cognitive Function

Lichtwark et al. investigated the relationship between cognitive function and mucosal healing in people with newly diagnosed CD following a GFD [42]. Eleven celiac patients (three men) between 22 and 39 years old followed a GFD for fifty-two weeks. During the intervention, several measurements such as blood and intestinal permeability tests, cognitive testing and dietary questionnaires were performed. Cognitive measurements were also completed at baseline, at week 12 and at the end of intervention, using the following tools: Subtle Cognitive Impairment Test (SCIT), Trail Making Test A & B, Controlled Oral Word Association Task (COWAT), Rey-Osterrieth Complex Figure (ROCF) immediate recall, Rey Auditory Verbal Learning Task (RAVLT), STPI, Grooved Pegboard Task, RAVLT delayed recall trial, Wechsler’s Test of Adult Reading (WTAR), and ROCF delayed recall trial. Results showed that after fifty-two weeks of following a GFD, Trail Making Task, SCIT and ROCF test performance improved significantly. However, no significant changes were observed in the rest of the cognitive tests and the STPI scale.

There is no ongoing clinical trial to verify the positive effects of a GFD observed in celiac patients. The NCT04296552 clinical trial, still in a recruiting status, will answer whether an LFD intervention could generate some type of cognitive effect.

#### 4.2.2. Potential Mechanism of Action in Cognitive Function

Due to their relation with the β-amyloid peptide or plaque formation, apoptosis, cell division, angiogenesis of neurovascular unit, tau protein aggregation or neuroinflammation, there are some genes and molecules that can be considered as potential biomarkers associated with AD, and consequently with cognition [64,87,88,89,90,91,92]. Among them is an enzyme that separates the amyloid beta A4 precursor protein from the beta zone (BACE2), FOXP3, NOTCH1, 4-cuppel factors (CLF4) PPARγ, chemokine ligand 2 (CXCL2) and calpain 13 (CAPN13). Some authors show that these markers are influenced by gluten. For example, in gluten-sensitive macaques, both BACE2 and CAPN13 levels were found to be higher [93]. In the same trials mentioned in the previous sections, altered levels of TGM2, FOXP3, NOTCH1 and PPARγ were observed in CD. In addition, CLF4 factor expression was reduced in paediatric CD [66,67,68,69].

As for emotional changes, systemic inflammation—marked by elevated cytokine levels—has also been associated with cognitive impairments. Gluten intake is able to modify the pro-inflammatory cytokine levels. However, it is necessary to highlight that its intake seems to raise cytokine levels in patients with CD, but not in those with self-reported gluten sensitivity [94]. Alternatively, it has been proposed that ES, which is closely related with amyloid fibres or insoluble aggregates including biomolecular condensates and EV from CD and NCGS patients, may exert an inflammatory effect in the brain promoting neurodegeneration [31].

Exposure to toxins can be related to neurodegenerative problems. An imbalance between the intestinal transport of essential and toxic metals can trigger later neurodegenerative processes [95]. In this sense, the expression of the divalent metals transporter 1 (DMT1) at the gut can play an important role, since it can transport toxic metals such as cadmium and lead. The relationship of this mechanism with gluten can be assumed in a study with CD patients, which presented higher levels of DMT1 mRNA and protein at the gut [96].

Animal studies have shown that gluten is able to decrease brain levels of tryptophan, and therefore serotonin. Taking into account the involvement of this neurotransmitter in cognitive processes, it cannot be discarded as a gluten consequence [54].

### 4.3. Schizophrenia

Schizophrenia is a psychiatric diagnosis that brings together severe chronic mental changes and is characterised by unusual behaviour and a modified perception of reality [97]. The classical classification of symptoms of this illness takes the form of three major categories [98]: positive symptoms (hallucinations, paranoia or distorted perceptions), negative symptoms (decrease in the ability to initiate plans, speak, express emotion or find pleasure) and disorganized symptoms (confused and disordered thinking and speech, trouble with logical thinking and sometimes bizarre behaviour or abnormal movements). Loss of cognition as well as mood symptomatology (depression) can also be involved with this mental illness.

#### 4.3.1. Gluten and FODMAP Intervention Trials in Schizophrenia

In the case of schizophrenia, only one clinical trial matched our review criteria. A randomised double-blind trial was conducted with sixteen schizophrenic or schizoaffective patients (18–64 years old; nine men) to observe the effects of the GFD on patients suffering from this disease [43]. Participants presented high levels of Antigliadin-IgG, although they were not celiac. The intervention consisted of adding milkshakes containing either I-gluten flour (10 g) or II-rice flour (10 g) to a GFD, thus separating two groups. Each group followed their diet for five weeks. For the study of positive symptoms, the BPRS test was used, designed to measure psychiatric disorders such as depression, anxiety, hallucinations and abnormal behaviour. Results showed no improvement in group II, due to the low effect size of the data. By contrast, in terms of SANS score, an improvement in negative symptoms was detected in patients on the rice flour diet. The SANS scale assesses negative symptoms in five blocks: affective impairment, alogia (communication problems), abulia/apathy, anhedonia (inability to enjoy) and frustration and attention disturbances. According to the scale score, Cohen’s d = −0.53 was the effect size. Taking into account that significant difference between the two groups was set at 0.50 in this trial, it concluded that gluten avoidance reduced symptomatology. Furthermore, the outcome of MATRICS Consensus Cognitive Battery (MCCB) showed an improvement of attention (Cohen’s d = −0.66) and oral memory (Cohen’s d = −0.37) in the gluten-free group. This survey assesses the seven cognitive domains affected by schizophrenia, as established by the US National Institute of Mental Health: speed of processing, attention, working memory, study and oral memory, study and visual memory, reasoning and occurrence and, finally, social cognition.

The lack of a larger sample number (7 participants followed GFD) in the only clinical evidence involving gluten and schizophrenia is, from our point of view, the limiting factor that could explain the inconsistent results obtained between SANS score and BPRS test by Kelly et al. [43], undermining the successful outcome observed for GFD in MCCB test.

The few trials addressing schizophrenia, and no trial assessing the potential effect of LFD in those patients, further restricted the encouraging results obtained in the study described. Furthermore, with the exception of one clinical test to assess GFD (NCT03183609), there is no other ongoing trial about this topic.

#### 4.3.2. Potential Mechanism of Action in Schizophrenia

Antipsychotics currently employed, especially second-generation antipsychotics, can cause side effects such as weight change or exacerbation of the cognitive problems associated with this mental disorder [99]. In this way, complementary therapies to pharmacological treatments are valued positively. Therefore, gluten restriction may also be beneficial as a parallel therapy for schizophrenia, according to the mechanisms proposed in the previous section on cognition.

It is also necessary to describe some genes and their possible effects on the disease. As in depression, PPARγ plays an important role in the pathogenesis of schizophrenia. Activation of PPARγ leads to an increase in the level of BDNF, which has a protective function in the nervous system, as mentioned above [64]. Thus, schizophrenia patients have shown a reduction in peripheral BDNF concentration.

As reported in the introduction, the relationship between gluten and schizophrenia is established. Different studies have shown that schizophrenic patients have registered high levels of antibody to gliadin [100,101]. Additionally, it has been documented that the permeability of the BBB can increase between 18 and 29% in patients with schizophrenia [102]. The linking point between both events may be zonulin, an intestinal tight junction modulator and Haptaglobunil2 precursor that can be triggered by gluten peptides [103]. Zonulin is expressed both in the human brain and in the intestine. Therefore, its over-expression could lead to an increase in permeability of the BBB.

Exposure to gluten by people with celiac disease and their subsequent development of specific antibodies can itself be considered a mechanism of action. Thus, gliadin specifics IgG and IgA have demonstrated the ability to bind synapsin I in celiac patients [104]. Synapsin I disruption has a marked implication on schizophrenia [105], which might explain the relationship of CD antibodies with schizophrenia since it promotes the release of neurotransmitters.

Exorphins have also been described as a possible mechanism of action of gluten on schizophrenia. Basic experimentation has shown that these peptides, which result from gluten digestion, can cross the BBB and bind to CNS opioid receptors, thus facilitating acquisition or consolidation of memory and learning processes [106]. Those data were complemented with the findings of different studies in schizophrenic patients where increased levels of small opioid-like neuroactive peptides were detected [107].

### 4.4. Autistic Spectrum

AS is a neurodevelopmental disorder involving impaired social communication and repetitive behaviours. It is characterised by depletion, oxidative stress and inflammation of glutathione in the brain, as well as neuroanatomical defects. Autism manifests during infancy and is characterised by limited verbal communication, lack of social interaction or responsiveness, and reduced, stereotyped and systematized patterns of interest and behaviour. The AS, in addition to autism, is a broader phenotype that includes less severe disturbances of agitation, such as Asperger’s syndrome [108].

#### 4.4.1. Gluten and FODMAP Intervention Trials in Autistic Spectrum

Regarding autism, three articles were collected. In the first, children with AS were engaged in a double-blind trial to detect whether a gluten- and/or casein-free diet affected the pathology. For this purpose, fourteen children with autism were assigned to one of these four diets: diet containing I—gluten, II—casein, III—gluten and casein and IV—neither gluten nor casein, i.e., placebo. During intervention, which lasted twelve weeks, in addition to the monitoring of diet and physiological functions, a behavioural study was performed using the Ritvo-Freeman test, which classifies behaviours related to the AS in children. At the end of the test, it was observed that the placebo group outcomes were similar to those of the group following the other diets, i.e., no significant differences were observed between the GFD and the gluten-containing diet. However, although not significant, some changes were observed in the Ritvo-Freeman test, specifically in the social relations and language sections. After individual analysis of these sections, no fixed pattern was observed, suggesting that those diets did not significantly influence behaviour [44].

Other research analysed the influence of a GFD on a group of sixty-six children (36–69 months old) with AS disorders [45]. Participants were divided into two groups: I—those who followed a GFD and II—those who consumed at least one normal meal containing gluten per day for six months. At baseline visit and at the end of the intervention each child underwent a comprehensive behavioural and psychometric assessment (i.e., the ADOS-2, the VABS-2, the SCQ, the ASRS, the Leiter scale). After six months, both the GFD group and gluten containing group improved the scores for ADOS-2 RRB domain, the SCQ score, and the ASRS tests. However, no differences were found between both groups.

By contrast, the research performed with children and adolescents suffering from AS by Ghalichi et al. [46], suggested a GFD as an effective treatment in controlling AS behaviours. Concretely, 80 AS patients were divided into two groups to follow a regular diet or GFD for 6 weeks. At the end of the intervention, the GFD group showed a reduction in Gilliam Autism Rating Scale 2 questionnaire score (GARS-2), in contrast with the regular diet group. Direct comparison between both diets disclosed significant differences in terms of stereotyped behaviours, communication and social interaction dimensions of GARS-2. For instance, after 6 weeks of treatment with a regular diet, stereotyped behaviours increased 2%, while a GFD diet promoted a reduction of 19%. In fact, in the stereotyped behaviours subscale, considerable improvement (*p* < 0.05) was observed for “eats foods”, “stares at hands objects”, “smells or sniffs objects”, “turns around self” or “sudden movements”.

Although only three studies involving GFD is a small number to draw reliable conclusions from, the characteristics and results of each of them seem to point to some evidences. In all studies, the trend or significant change after the GFD intervention always emerged in social symptoms. Only the study by Ghalichi et al. registered a general improvement in the questionnaire used to measure autism-related symptoms (GARS-2) [46]. Thus, the GFD intervention also produced beneficial changes in behaviour and communication domains.

It is worth noting that in the research related to young children (3–6 years old), one study of short duration (2 weeks) [44] and another of medium-long duration (6 months) [45], there were no changes between the GFD group and the control. On the contrary, in the medium-duration intervention (6 weeks) carried out with children and adolescents aged between 4 and 16 years, the GFD group showed a general improvement in the overall score of AS symptoms [46]. Therefore, it could be speculated that the age of the patients suffering from AS is a key factor for making GFD follow-up effective against the social symptoms of AS.

In regard to FODMAP effects, Nogay et al. conducted a pilot study to assess the effect of a low FODMAP diet on gastrointestinal and behavioural problems in children with AS disease [47]. Fifteen children (6–17 years old) who reported constipation and/or abdominal pain were enrolled in this study. Two groups were randomly defined in which seven children followed a LFD and eight children (control group) kept to a normal diet for two weeks. Behavioural problems were assessed at baseline and after two weeks by the Aberrant Behaviour Checklist-Community (ABC-C). This checklist has five domains: (I) irritability; (II) social withdrawal; (III) stereotypic behaviour; (IV) hyperactivity and (V) inappropriate speech. Comparing the results of the domains before and after the intervention, irritability scores tended to reduce (*p* < 0.1) in the LFD group at the follow-up, even if it was not statistically significant (*p* < 0.05). By contrast, the hyperactivity/noncompliance score was significantly higher in the control group compared with the baseline. Furthermore, social withdrawal, stereotypic behaviour, and hyperactivity/noncompliance tended to be reduced in LFD compared with the habitual diet group, but once again, no significant difference was observed (*p* < 0.1).

Authors concluded that more studies are necessary, including larger samples to confirm the effects of LFD in children with autism who have gastrointestinal problems. However, there is no other trial (neither finished nor ongoing) about the topic. After the reviewing processes, doubts about FODMAP implication in the AS can be extended to gluten due to the lack of robust results.

#### 4.4.2. Potential Mechanism of Action in Autistic Spectrum

The AS can be understood in a general way as a set of behavioural abnormalities according to age, with cognition and emotions being two key elements in its development [109]. For this reason, we can find a relationship in this disease with the mechanism of almost all the previous sections (Section 4.1.2, Section 4.2.2 and Section 4.3.2). A similar pattern is found in the pharmacological treatments proposed for the AS, where antidepressants and antipsychotics play a relevant role. In this way, Section 4.4.2 also contains interesting mechanisms concerning this pathology. Thus, bacterial dysbiosis, intestinal permeability, inflammation, exorphines, or CNS neurotransmitter reduction are interesting mechanisms for the treatment of the AS. These in turn may constitute the mechanisms of action by which FODMAPs or gluten exert their effect. For instance, girls and women with Rett syndrome (classified within the AS) and normal values of endomysium or trans-glutaminase antibodies, reported higher values of IgG and IgA against gliadins. This fact may indicate a change in intestinal permeability linked to an inflammatory activation in autistic patients, without constitutive CD markers [110].

At this point, the gluten degradation paradox must be explained. The complete degradation of gliadin contributes to the lack of gluten epitopes that possess inflammatory activity in sensitive people [2]; a clear negative aspect. Notwithstanding, incomplete degradation due to inactivity or deficiency of the enzyme dipeptidyl peptidase IV (DPP IV) can lead to the appearance of opioid-like peptides called exorphines; a clear positive aspect in the treatment of the AS. It is interesting to note that gliadin has an inhibitory effect on the DPP IV enzyme. Like casein from dairy products, gliadin itself has a very high affinity for DPP IV; even higher than that for substance P, an endogenous substrate of the enzyme [111]. Taking into account that substance P has been involved with neurological, inflammatory and endocrine processes, its degradation disturbance may be responsible for disorders related to the aforementioned processes [112].

Another protein that is not only related to autism, but also to Schizophrenia and AD is C1Q [112]. Since it is responsible for binding and eliminating immunoglobulin complexes bound to antigens, C1Q is a protein involved in neuronal remodelling of the CNS. Moreover, it has been proposed that C1Q binds preferentially to immunoglobulins coupled with casein and gluten antigens [113]. Nevertheless, it cannot be discarded that the inhibition of DPP IV generated by gliadin exorphins promotes C1Q overexpression [112].

In the particular case of FODMAP and inflammation, it seems that not all molecules have the same effect, at least in IBS. According to a study carried out in twenty patients with IBS and diarrhoea, after following a LFD diet for nine weeks, serum pro-inflammatory cytokines IL-6 and IL-8 were decreased [114]. By contrast, after incorporating FOS into the diet for ten days, cytokine levels did not change in the patients. Reinforcing the idea that FOS does not have the same effect, SCFAs were measured in the same study. Although SCFAs were significantly lower after the LFD, no changes took place once FOS was supplemented to the diet. It is necessary to remember that SCFA increases PYY secretion, and influences memory and learning processes [115]. Related also with the inflammatory processes, C-reactive protein (CRP) has been suggested to contribute to cognitive decline [116]. A meta-analysis has shown that fermentable oligosaccharides (FOS, GOS) reduce CRP levels [117].

In general terms, the fructans subgroup, including FOS, differs from the rest of the FODMAPs. Apart from the immunomodulatory effect of fructans [118], according to a study conducted with obese mice, extracts of fructans and oligofructose from agave reduce two biomarkers of oxidative stress—thiobarbituric acid reactive substances (TBARS) and protein carbonyl levels—in brain areas related to cognitive processes such as the hippocampus, frontal cortex and cerebellum [119]. Oxidative stress has been recently proposed as a possible mechanism of cognitive decline [120].

## 5. Conclusions

In view of these results, it seems that avoiding gluten or reducing its consumption might be beneficial to improve the symptoms of depression, anxiety and cognition in people with IBS, CD and Fibromyalgia. However, the effect of a LFD on the development of these symptoms is more uncertain. Although results indicate that a decrease in the consumption of these compounds brings about an improvement in anxiety and depression, more studies are necessary in order to reach more solid conclusions. We believe that the clinical trials that are currently ongoing will be a step forward in this direction.

With regard to mental diseases, in the trial on Schizophrenia it was observed that avoiding gluten can lead to an improvement in cognition. In the case of the AS, the exclusion of gluten or reducing FODMAPs from the diet may be beneficial, although not in a generalised and significant way. While studies in this field are scarce and more evidence is necessary to come to stronger conclusions, these studies open the door to future research linking diet to mental diseases.

## Figures and Tables

**Table 1:** Characteristics and results of clinical and randomized controlled trials related to depression, anxiety and cognitive mental symptoms.

Mental Symptom	Reference	Study Design and Participants	Intervention	Outcomes Related to Mental Symptom	Results
Depression/anxiety	[24]	Randomized double-blind, crossover study *n* = 22 Male = 5 Female = 17 Non-celiac irritable bowel syndrome patients 24–62 years old	Three diets: - (I): gluten (16 g/day) - (II): whey (16 g/day) - (III) not supplemented (placebo) All participants follow for 3 days each diet (3–14 days wash-out period between each)	Spielberger State Trait Personality Inventory (STPI): - State indices - Trait indices	Comparison among intervention groups: - ↑ STPI state depression score with gluten diet vs. placebo - No changes for STPI state anxiety score - No changes for trait indices
Depression/anxiety	[38]	Randomized not blinded study *n* = 40 Male = 26 Female = 14 Symptomatic adults with endomysial antibodies 21–74 years old	Two diets: - (I) Gluten-free diet (GFD) (*n* = 20) - (II) Normal diet (containing gluten) (*n* = 20) Each group follow their diet for 12 months	General Well-Being (PGWB): Including anxiety, depression dimensions	Comparison among intervention groups: - No differences between treatment groups in depression dimension - Magnitude of improvement in anxiety scores was greater for GFD
Depression	[37]	Randomized double-blind, crossover study *n* = 61 Male = 8 Female = 53 Suspected non-celiac gluten sensitivity patients	Two groups: - (I) Gluten-containing diet (4.375 g/day gluten) - (II) Placebo diet All participants follow for 1 week each diet (wash-out period of 3 weeks after each)	Non-validated rating scale depression questionnaire State-Trait Anxiety Inventory (STAI)	Comparison before and after the intervention: - Gluten ↑depression - Placebo did not change STAI state or trait - Comparison among intervention groups: - Gluten worsened depression symptoms and placebo did not
Depression/anxiety	[39]	Randomized non-blinded study *n* = 74 Male = 14 Female = 60 Irritable bowel syndrome (IBS) patients 20–72 years old	Three groups: - (I): Hypnotherapy (*n* = 25) - (II): Low FODMAP diet (LFD) (*n* = 24) - (III): combination of I and II (*n* = 25) Each group follow their diet for 6 weeks	Hospital anxiety and depression Scale (HADS) STPI: - State indices - Trait indices	Comparison before and after the intervention: Short-term (6 weeks): - NS changes in anxiety and depression for STPI state or trait indices with LFD - LFD ↓ anxiety and depression according to HADS Long-term (6 months): - NS changes in anxiety and depression for STPI state or trait indices with LFD - LFD ↓ anxiety but not depression according to HADS Comparison among intervention groups: - No differences across treatment groups
Depression/anxiety	[36]	Double blinded study *n* = 41 Male = 10 Female = −31 IBS and diarrhoea patients	One diet: - (I) GFD Two groups: - HLA-DQ − (*n* = 21) - HLA-DQ + (*n* = 20) Each group follow a GFD for 6 weeks 21 patients follow the GFD for 18 months	HADS	Comparison before and after the intervention: - GFD ↓ HADS at 6 weeks and 18 months
Depression/anxiety	[40]	Randomized non-blinded study *n* = 75 Male = 2 Female = 73 32–66 years old Patients with fibromyalgia	Two diets: - (I) GFD (*n* = 35) - (II) Hypocaloric diet (*n* = 40) Each group follow their diet for 6 months	Beck Depression Inventory-II (BDI-II) STAI	Comparison before and after the intervention: - GFD ↓ BDI-II - GFD NS change in STAI state or trait Comparison among intervention groups: - No differences across treatment groups
Depression/anxiety	[41]	Randomized single-blinded study *n* = 92 Male = 27 Female = 65 19–75 years old IBS and diarrhoea patients	Two diets - (I): LFD (*n* = 50) - (II): healthy diet (*n* = 42) Each group follow their diet for 4 weeks	HADS	Comparison before and after the intervention: - LFD ↓ depression - LFD ↓ anxiety Comparison among intervention groups: - Magnitude of improvement in anxiety scores was greater for LFD
Cognition	[42]	Longitudinal study *n* = 11 Male = 3 Female = 8 Celiac patients 22–39 years old	All participants following a GFD for 52 weeks	Cognition measurements: - Subtle Cognitive Impairment Test (SCIT) - Trail Making Test A & B - Controlled Oral Word Association Task (COWAT) - Rey-Osterrieth Complex Figure (ROCF) - Rey Auditory Verbal Learning Task (RAVLT) - Grooved Pegboard Task - Wechsler’s Test of Adult - Reading (WTAR) Other measurements - STPI	Comparison before and after the intervention: - Trail Making Task, SCIT and ROCF test performance improve after the intervention - NS changes were observed in the rest of cognitive test and STPI scale

NS: not significant.

**Table 2:** Characteristics and results of clinical and randomized controlled trials related with schizophrenia and autism spectrum mental diseases.

Mental Symptom	Reference	Study Design and Participants	Intervention	Outcomes Related to Mental Symptom	Results
Schizophrenia	[43]	Randomized double-blind, study *n* = 16 Male = 9 Female = 7 18–64 years old Non-celiac schizophrenic or schizoaffective patients	Two diets - (I):Gluten-containing diet (GD) = Gluten-free diet (GFD) + gluten flour (10 g) (*n* = 9) - (II): GFD = GFD + rice flour (10 g) (*n* = 7) Each group follow their diet for 5 weeks	Psychiatric symptoms measurement - Positive symptoms by using Brief Psychiatric Rating Scale (BPRS) - Negative symptoms using Scale for the Assessment of Negative Symptoms (SANS). - Calgary Depression Scale (CDS) - Clinical Global Impression scale (CGI) Cognitive function measurement: Measurement and Treatment Research to Improve Cognition in Schizophrenia (MATRICS) consensus Cognitive Battery (MCCB)	Comparison before and after the intervention: - No change in BPRS, CDS and CGI following both diets - GFD ↓ SANS Comparison among intervention groups: - GFD ↓ MCCB comparing with GD
Autism spectrum	[44]	Randomized double-blinded study *n* = 14 Male = 12 Female = 2 3–5 years old Patients with autism spectrum disorders	Four snacks Each participant received weekly snack that contain - (I): gluten - (II): casein - (III): casein and gluten - (IV): placebo 12 weeks of dietary challenge (1 day/week). Divided in 3 blocks, which contain the 4 snacks (1 per week) 30 weeks of total follow-up	Ritvo-Freeman Real Life Rating Scales	Comparison among intervention groups: - Social relationship symptoms and language symptoms of Ritvo-Freeman Real Scales tended to reduce in gluten group comparing with placebo group (statistically not significant) - No changes were observed in sensory motor, affectual reactions and Sensory responses of Ritvo-Freeman Real Scales across treatment groups
Autism spectrum	[45]	Randomized single-blind, study *n* = 66 Male = 56 Female = 10 3–6 years old Patients with autism spectrum disorders	Two diets - (I):GD = GFD + one meal with gluten (*n* = 33) - (II): GFD (*n* = 33) Each group follow their diet for 6 months	Autism symptoms measurement: Autism Diagnostic Observation Schedule, Second Edition ADOS-2 - Social affect domain (–SA) - Restricted and repetitive behaviours domain (-RRB) Autistic symptoms assessed by parents: - Social Communication Questionnaire (SCQ) - Autism Spectrum Rating Scale (ASRS))	Comparison before and after the intervention: - Improvements in the ADOS-2 RRB domain score, the SCQ score, and the ASRS Total Score in both groups Comparison among intervention groups: - No differences across treatment groups
Autism spectrum	[46]	Randomized single-blind, study *n* = 80 (4 out) Male = 56 Female = 20 4–16 years old Patients with autism spectrum disorders	Two diets - (I):GD = regular diet (*n* = 38) - (II): GFD (*n* = 38) Each group follow their diet for 6 weeks	Autism symptoms measurement: Gilliam Autism Rating Scale 2 questionnaire (GARS) - Stereotyped behaviours domain - Communication domain - Social interaction domain	Comparison before and after the intervention: - Improvements in the GARS-2 total score as wells as stereotyped behaviours, communication and social domain interaction in GFD group. - In GD group only improved social interaction domain. Comparison among intervention groups: - GFD ↓ stereotyped behaviours, communication and social domains comparing with GD
Autism spectrum	[47]	Randomized not blinded study *n* = 15 Male = − Female = − 6–17 years old Patients with autism spectrum disorder	Two diets: - (I): Low FODMAP diet (LFD)(*n* = 7) - (II): normal diet (*n* = 8) Each group follow their diet for 2 weeks	Aberrant Behaviour Checklist-Community including 5 domains: - Irritability - Social withdrawal - Stereotypic behaviour - Hyperactivity - Inappropriate speech	Comparison before and after the intervention: - Irritability scores tended to reduce in the LFD group at follow-up compared with the baseline (statistically not significant) - The hyperactivity/noncompliance score was significantly higher in the control group at follow-up compared with the baseline Comparison among intervention groups: Social - Withdrawal, stereotypic behaviour, hyperactivity/noncompliance tended to reduce in LFD comparing with normal diet group (statistically not significant)

Tendency *p* < 0.1; significance was stated at *p* < 0.05.

## Data Availability

Not applicable.
